# Supplementary material for: Application of Autologous Peripheral Blood Mononuclear Cells into the Area of Spinal Cord Injury in a Subacute Period: A Feasibility Study in Pigs
Source: Biology (Basel). 2021 Jan 24;10(2):87. doi: 10.3390/biology10020087 (PMC7911660; doi:10.3390/biology10020087)
Supplement: Supplementary file 1 [file biology-10-00087-s001.pdf]

Table S1. Blood and cerebrospinal fluid cytokine/chemokine concentrations (ng/mL) on days 7 and 14 after FM or FM+PBMCs application. * p<0.05 when compared with similar analytes of intact samples, # p<0.05 when compared with similar analytes in this group in 14 days after therapy. Text in bold indicates significant difference (p<0.05) in this analyte between these groups in the corresponding period after therapy. < - protein concentrations below the specified value could not be detected with the kit.

cytokine/ chemokine		Intact control	7 days after FM/ FM+PBMCs	14 days after FM/ FM+PBMCs
GM-CSF	blood	0.011±0.003	0.02±0.01/0.09±0.05*	0.01±0.005/0.11±0.09*
	CSF	0.006±0.003	0.01±0.006*/0.02±0.005*	0.016±0.005*/0.013±0.005*
IFN-g	blood	22.78±7.03	**7.16±6.34/25.54±4.46#**	8.99±5.35/15.81±2.61
	CSF	<0.12	0.79±0.47/1.05±0.17	1.12±0.05/0.63±0.35
IL-10	blood	0.05±0.02	0.19±0.15/0.44±0.47*	0.05±0.04/0.26±0.2*
	CSF	0.008±0.0007	<0.01/0.23±0.005*	<0.01/<0.01
IL-12	blood	0.01±0.007	0.21±0.16*/0.49±0.08*#	0.3±0.12*/0.72±0.14*
	CSF	0.009±0.0008	<0.01/0.07±0.01*	0.1±0.005*/0.6±0.3*
IL-18	blood	0.24±0.1	0.29±0.2/0.81±0.55*	**0.16±0.1/0.69±0.25***
	CSF	0.025±0.022	**0.29±0.3*/2.65±0.12***	0.09±0.01/0.09±0.01
IL-1a	blood	0.11±0.15	<0.01/0.06±0.03	0.01±0.005/0.03±0.01
	CSF	<0.008	<0.01/0.01±0	<0.01/<0.01
IL-1b	blood	0.29±0.32	0.03±0.007/0.04±0.02	0.03±0.015/0.04±0.005
	CSF	<0.009	0.02±0.005/0.04±0.005	0.02±0/0.02±0.005
IL-1Ra	blood	<0.01	0.15±0.09/0.22±0.05	0.08±0.03/0.62±1
	CSF	<0.01	**1.28±0.17#/0.62±0.02**	0.95±0.06/0.59±0.72
IL-2	blood	0.41±0.66	0.08±0.07/0.17±0.16	<0.01/0.16±0.05
	CSF	0.015±0.005	<0.01/<0.01	<0.01/<0.01
IL-4	blood	0.25±0.24	0.1±0.09/0.68±1.06	0.03±0.01/0.3±0.26
	CSF	<0.009	0.01±0.005/0.02±0	0.01±0/0.02±0.01
IL-6	blood	0.52±0.2	0.025±0.021*/0.047±0.042*	0.006±0.005*/0.05±0.06*
	CSF	0.03±0.012	0.02±0.005/0.07±0.1	0.016±0.005/0.01±0.005
IL-8	blood	0.6±0.42	0.1±0.02/0.18±0.08	0.01±0.005*/0.51±0.58*
	CSF	0.07±0.04	0.01±0.005/0.03±0.01	0.016±0.005/0.04±0.02
TNF-a	blood	0.17±0.19	0.2±0.03/0.15±0.06	0.03±0.02/0.15±0.06
	CSF	0.01±0.008	<0.01/0.023±0.005	<0.01/<0.01
